# Development of a TaqMan-based multiplex real-time PCR for simultaneous detection of four feline diarrhea-associated viruses

**DOI:** 10.3389/fvets.2022.1005759

**Published:** 2022-11-03

**Authors:** Junwei Zou, Ju Yu, Yuanyuan Mu, Xiangyu Xie, Run Wang, Haiqiang Wu, Xuan Liu, Fazhi Xu, Juhua Wang, Yong Wang

**Affiliations:** College of Animal Science and Technology, Anhui Agricultural University, Hefei, China

**Keywords:** TaqMan-based multiplex qPCR, detection, co-infection, FPV, FBoV-1, FeAstV, FeKoV

## Abstract

Since their recent discovery, the prevalence of novel feline enteric viruses, including feline bocavirus 1 (FBoV-1), feline astrovirus (FeAstV), and feline kobuvirus (FeKoV), has been reported in China. Co-infections of these viruses with feline parvovirus (FPV) are common causes of diarrhea in cats. Viral co-infections are difficult to identify because of their non-specific clinical signs. To detect and identify these viruses, a quick and specific pathogen-testing approach is required. Here, we establish a real-time PCR (qPCR) based on multiple TaqMan probes for the simultaneous detection of FBoV-1, FeAstV, FeKoV, and FPV. Specific primers and TaqMan fluorescent probes were designed to ensure specificity. The results showed that the detection limit of single qPCR was up to 10 copies, and the detection limit of multiplex qPCR was up to 100 copies, with correlation coefficients >0.995 in all cases. Clinical sample detection revealed a 25.19% (34/135) total rate of co-infection among the viruses and a 1.48% (2/135) quadruple infection rate. Thus, this multiplex qPCR approach can serve as a quick, sensitive, and specific diagnostic tool for FBoV-1, FeAstV, FeKoV, and FPV identification, and it may be utilized for routine surveillance of these emerging and reemerging feline enteric viruses.

## Introduction

Cats are the second most common pets in the world; with them being an important member of many families, feline health has become increasingly valued (1). However, cats are prone to viral diarrhea (2), and enteric viruses are always co-infected in cats with diarrhea (3–5). While feline parvovirus (FPV) is considered an important cause of diarrhea (6), more emerging feline viruses have recently been reported, including feline bocavirus 1 (FBoV-1) (4), feline astrovirus (FeAstV) (5), and feline kobuvirus (FeKoV) (7). Because of the high probability of co-infection and the relative similarity between clinical symptoms caused by these four viruses, some of the viruses are frequently missed in clinical diagnosis and epidemiological research. Thus, the need for the development of detection technologies for these viruses is becoming increasingly urgent.

FPV is a small, nonenveloped ssDNA virus belonging to the genus *Protoparvovirus* within the family *Parvoviridae* (8, 9). It is a member of the Carnivore *protoparvovirus* 1 and is highly infectious and usually infects kittens within 6 months of age via the fecal-oral route (2). FPV rapidly damages replicating cells in the crypts of the intestinal mucosa, eventually resulting in diarrhea caused by malabsorption and increased permeability. FPV is a widespread epidemic that seriously endangers the health of cats and can cause immunosuppression, which subsequently induces co-infection from other feline viruses (6). Feline bocavirus (FBoV) is an ssDNA virus that belongs to the Carnivore *bocaparvovirus* group of the genus *Bocaparvovirus* (9). FBoV was first discovered and reported in Hong Kong in 2012 (10). Three genotypes of FBoV have been identified: FBoV-1, FBoV-2, and FBoV-3, which correspond to Carnivore bocaparvovirus-3, 4, and 5 (1, 9, 11). The first report on the prevalence and genetic diversity of FBoV-1 in northeast China was published in 2018 (3). In 2019, Piewbang et al. reported that FBoV-1 was associated with outbreaks of hemorrhagic enteritis in household cats. This provided direct evidence that FBoV-1 causes intestinal infection and revealed its pathological effects in domestic cats, suggesting that FBoV-1 and FPV co-infection may lead to more severe clinical manifestations (4). The family *Astroviridae* comprises small non-enveloped viruses with a positive ssRNA genome. It contains a single genus, Astrovirus, which comprises all astroviruses (12). FeAstV is a common feline enterovirus. Cats with diarrhea also infected with FeAstV have been reported in Italy (13), Japan (14), Australia (5), and China (15, 16). FeAstV is associated with diarrhea in cats. FeKoV is a small, spherical, non-enveloped virus with a single-stranded, positive-sense RNA genomes (17). It is classified into the genus *Kobuvirus* within the family *Picornaviridae* in 1999. FeKoV was first detected in diarrheal cat feces in South Korea in 2013, and was first reported in China in 2018 (7, 18). Co-infections of FeKoV with FPV and Feline enteric corona virus (FECV) were reported by Di Martino et al. who suggested that FeKoV, as a novel virus, is a common component of feline enteric viruses (19, 20). FeKoV is circulating in China, with an infection rate of 14.2% (28/197) (21). To date, no RT-qPCR is available for detection of FeKoV.

TaqMan qPCR provides high sensitivity and specificity for quantitative analysis. Currently, only FPV, FeAstV, and FBoV-1 have established TaqMan qPCR detection methods (22–24), and any associated testing methodologies for FKoV have yet to be reported. Zhang et al. established multiplex PCR assays for diarrhea-associated viruses in cats, including FPV, FBoV-1, and FeAstV (2). In this study, we aimed to develop and evaluate a multiplex TaqMan qPCR technology for the quantitative and differential detection of FBoV-1, FeAstV, FeKoV, and FPV in the feces of cats with diarrhea.

## Materials and methods

### Virus strains and clinical samples

All the viral strains utilized in this study were isolated from clinically positive samples, and the extracted nucleic acid was stored at −20°C until needed. The viral sequences were uploaded to GenBank after identification by the General Biology Company (Anhui, China). Nucleic acids were extracted from the FPV HF1 strain (GenBank accession number: MT614366), FBoV-1 AAU01 strain (MT577646), FBoV-2 HFXA-6 strain (MT633126), FBoV-3 HFXA-18 strain (MT633128), FeAstV AH-1-2020 strain (MN977118), FeKoV HFZ-FKV1 strain (ON219928), feline coronavirus (FCoV) HF1902 strain (MT444152), and feline chaphamaparvovirus (FeChPV) HF2 strain (MT708231).

Clinical samples were obtained from Anhui, Jiangsu, Shanghai, and Guangdong provinces between 2018 and 2021. A total of 135 clinical samples (stool samples from cats with diarrhea symptoms) were collected from various veterinary hospitals and rescue stations and kept at −20°C in the laboratory before use.

### Nucleic acid extraction

Fecal samples were treated with PBS, and the supernatant was collected. Both DNA and RNA were extracted from the samples according to the manufacturer's instructions using the TIANamp Virus DNA/RNA Kit (Tiangen, Beijing, China) and stored at −20°C until use. The extracted total RNA was transcribed into cDNA using the FastKing RT Kit (with gDNase) (Tiangen, Beijing, China) according to the manufacturer's instructions. All DNA and cDNA were stored at −20°C until use.

### Design of specific primers and probes

All available complete sequences of FBoV-1, FeAstV, FeKoV, and FPV were collected from GenBank. After comparison, the conservation of genes was determined for designing the primers and probes. Four sets of specific primers and corresponding probes were designed using the Primer Premier 5 software. Potential cross-reactivity and target specificity were detected and examined using the Basic Local Alignment Search Tool. Primers and probes were synthesized by General Biology Company (Anhui, China). The details are presented in Table 1.

**Table 1:** Primers and probes designed for the multiplex qPCR.

**Virus**	**Primer/probe**	**Sequence (5^′^–3^′^)**	**Size (bp)**	**Target gene**	**References**
FeAstV	F	GCTTCGTGACTCTGGGCTTC	137	ORF1b	(16)
	R	TCGGCCATTGGTGTTATTGAC			
	Probe	Hex-TGGAGGGGAGGACCAAAAGACTGTAATG-BHQ1			
FBoV-1	F	GCGGTGGTCACTCACAGGAT	114	NS1	(23)
	R	CTGGTGTAGCGTCCTCGATGT			
	Probe	CY5-CGGAACGTGTATGAGCTGTGGCGG-BHQ2			
FeKoV	F	AACGCCCTGGAAAGAGTGAA	112	5' UTR	This study
	R	GAAATGAAACTACCCGTTAGACAAT			
	Probe	FAM-TTCTACTGCCCTAGGAATGCCACGC-BHQ1			
FPV	F	GAACAAATGAAACCAGAAACCGT	126	NS1	This study
	R	TCTTTTACTAACCAAGTCCCGCA			
	Probe	TexasRed-ACCGTTGAAACCACAGTGACGACAGCA-BHQ2			

### Preparation of standard plasmids

Target fragments of FBoV-1, FeAstV, FeKoV, and FPV were amplified by PCR, using the same primers as those used for multiplex qPCR. The total reaction volume was 20 μL, including 10 μL of 2 × Taq PCR MasterMix II, 250 nmol forward and reverse primer, and 1 μL of template DNA, with double-distilled water [ddH_2_O] added to the constant volume of 20 μL. The thermal cycling procedure was as follows: 94°C for 5 min, 40 cycles of 94°C for 30 s, 60°C for 30 s, 72°C for 30 s, and 72°C for 10 min. Distilled water was used as a negative control. Amplification was performed on a Hema96TC instrument. PCR products were separated by agarose gel electrophoresis, and the TIANgel Midi Purification Kit was used for gel recovery of nucleic acids linked to the pMD19-T vector and transferred to *E. coli* dH5α receptor cells. Positive clones were cultured, their plasmids were extracted, and their concentrations determined. The constructed plasmid was confirmed by the General Biology Company (Anhui, China).

The copy number of the extracted plasmids was calculated according to the following formula.


$$\begin{array}{l} \text{Plasmid copies}/\text{μL}=\frac{\left(6.02\;\times \;10^{23}\right)\;\times \;\left(X\;ng/\mu L\;\times \;10^{-9}\right)}{\text{plasmid length }\left(\text{bp}\right)\;\times \;660} \end{array}$$

The plasmid copy number was calculated from 10^8^ copies/μL to 10^1^ copies/μL using a 10-fold diluted plasmid for single qPCR of each virus to generate four standard curves. The standard equation was calculated according to the E value (amplification efficiency) and R^2^ value (correlation coefficient).

### Optimization of the single and multiplex TaqMan qPCR assays

All qPCR reaction systems were calibrated at 20 μL. Amplification was performed using LightCycler^®^ 96.

The single qPCR system comprised 10 μL of 2 × TaqMan Fast qPCR Master Mix, 200 nmol forward and reverse primers, 100 nmol probe, and 1 μL of template DNA, with ddH_2_O added to ensure a uniform volume of 20 μL. Multiplex qPCR was conducted after several optimizations of the reaction system composition to determine the optimal multiple reaction system.

The thermal cycling procedure was as follows: 94°C for 3 min, 40 cycles of 95°C for 5 s, and 60°C for 30 s. Signals were automatically collected at the end of each cycle.

### Sensitivity test of the multiplex qPCR assay

The optimized multiplex reaction system was used for multiplex qPCR experiments. The final concentration of each viral plasmid ranged from 10^8^ to 10^1^ copies/μL as a template to determine the detection limit of the multiplex qPCR. Three replicates were used for each concentration.

### Specificity test of the multiplex qPCR assay

To avoid false positives caused by other viruses that may be present in the assay, the multiplex qPCR detection technology was used to detect FBoV-2, FBoV-3, FCoV, and FeChPV.

### Repeatability test of the multiplex qPCR assay

The standard plasmid templates were used in the multiplex qPCR and data acquisition. To ensure the repeatability of the detection method, all templates were repeated three times. The experiment was repeated with different participants over 3 days, and the coefficients of variation were calculated by intra-assay and inter-assay analyses.

### Examination of clinical fecal samples using the multiplex qPCR

From July 2018 to September 2021, fecal samples from 135 cats were collected from pet hospitals and rescue stations in the Anhui, Jiangsu, Shanghai, and Guangdong provinces of China from cats with diarrhea symptoms. All samples were tested using a multiplex qPCR assay to determine virus positivity of the samples. To ensure the authenticity and reliability of the procedure, all positive samples were sequenced by the General Biology Company (Anhui, China).

The infection rate of each virus was analyzed after obtaining the assay results of all clinical samples.

## Results

### Optimized single qPCR for individual viruses

**Figure 1:**
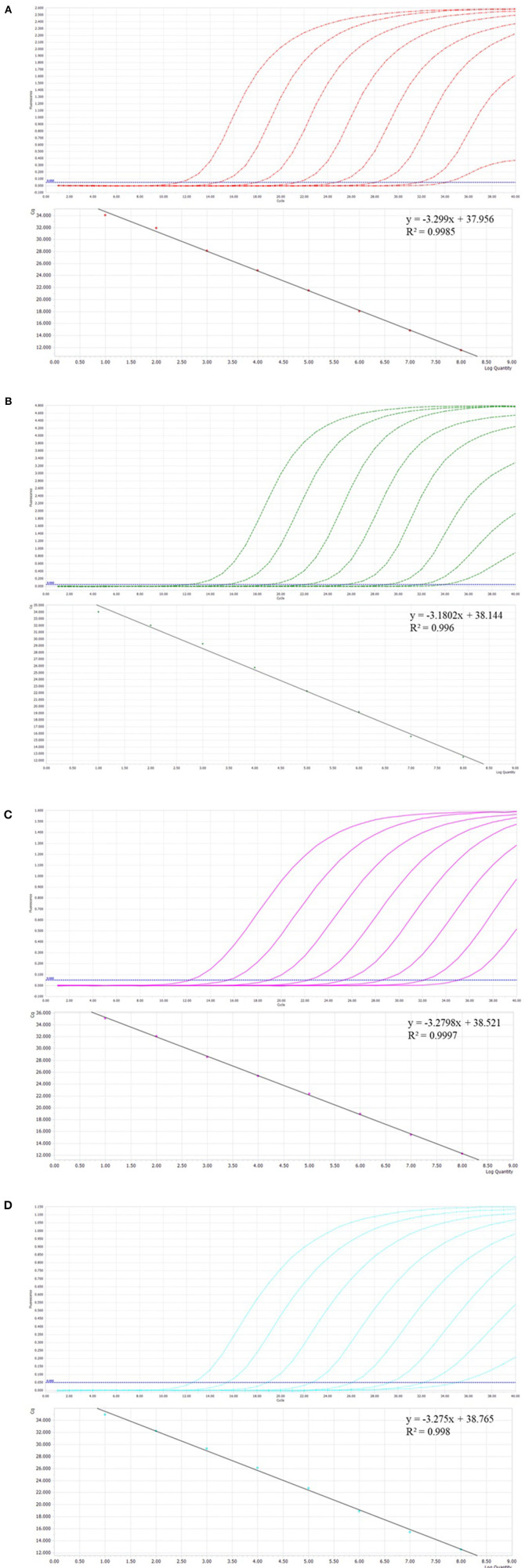
**(A–D)** The amplification curves (top, X-axis: Cycle, Y-axis: Fluorescence) and standard curve (bottom, X-axis: Log Quantity, Y-axis: Cq) for the single qPCR assay of individual viruses. **(A)** Feline parvovirus (FPV) plasmids from 10^8^ to 10^1^ copies/μL. **(B)** Feline bocavirus 1 (FBoV-1) plasmids from 10^8^ to 10^1^ copies/μL. **(C)** Feline kobuvirus (FeKoV) plasmids from 10^8^ to 10^1^ copies/μL. **(D)** Feline astrovirus (FeAstV) plasmids from 10^8^ to 10^1^ copies/μL.

Prior to the development of multiplex assays, a single qPCR was established for each virus. Standard curves were established for each virus using 10-fold serial dilutions of linear plasmids from 10^8^ to 10^1^ copies/μL. The standard curves showed good amplification efficiency, with the correlation coefficients of FBoV-1 (R^2^ = 0.996; Eff% = 107), FeAstV (R^2^ = 0.998; Eff% = 102), FeKoV (R^2^ = 0.997; Eff% = 102), and FPV (R^2^ = 0.9985; Eff% = 101). The quantification cycle (Cq) values of the single qPCR assay for the individual viruses are shown in Table 2. All primers and probes designed using this technology were efficient and sensitive, and all plasmids were qualified, as shown in Figure 1. The minimum effective positive fluorescence (EPF) was set at 0.05 according to the instrument's default. The Cq value was recorded when the EPF was 0.05, and Cq values >35 were considered negative.

**Table 2:** The quantification cycle (Cq) values of the single qPCR assays for individual viruses.

**Templates (copies/μL)**	**FPV**	**FBoV-1**	**FeKoV**	**FeAstV**
10^8^	11.52	12.48	12.22	12.56
10^7^	14.81	15.55	15.44	15.46
10^6^	18.06	19.19	18.95	18.92
10^5^	21.48	22.29	22.33	22.69
10^4^	24.86	25.78	25.36	26.12
10^3^	28.15	29.3	28.58	29.3
10^2^	31.92	32.03	32.11	32.22
10^1^	34.08	34.04	35.11	34.95

### Optimized multiplex qPCR

In the multiplex qPCR, the primers and probes for different viruses interacted with each other. In this experiment, four fluorescence groups with different excitation wavelengths were selected for the design of separate probes for each virus to ensure the normal recognition of the instrument. The optimal concentration of primers and probes was determined by optimizing the reaction system, with a final reaction volume of 20 μL (10 μL 2 × TaqMan Fast qPCR Master Mix, 800 nmol forward and reverse primers, 400 nmol probe, and 1 μL template of viruses, with ddH_2_O added until the constant volume of 20 μL was reached). The thermal cycling procedure was as follows: 94°C for 3 min, 40 cycles of 94°C for 5 s, and 60°C for 30 s. Signals were automatically collected at the end of each cycle.

**Figure 2:**
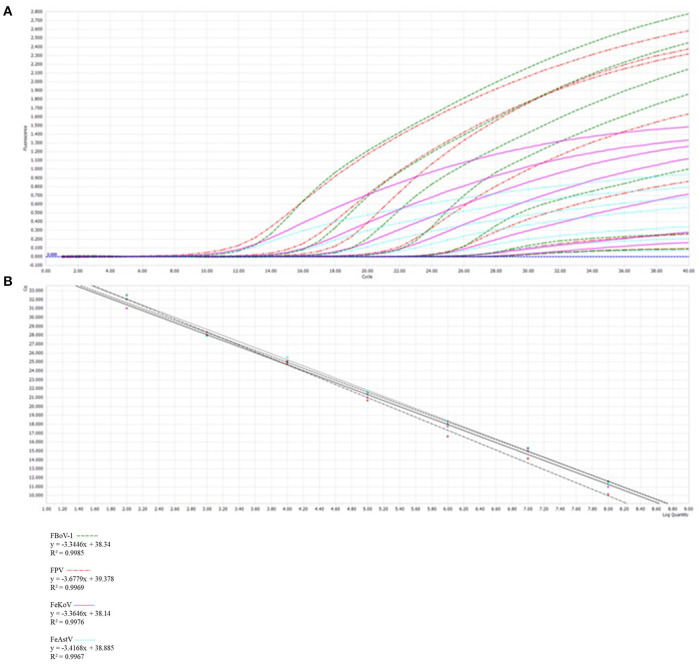
Development of a multiplex qPCR detection method. **(A)** The amplification curves (top, X-axis: Cycle, Y-axis: Fluorescence) and standard curve (bottom, X-axis: Log Quantity, Y-axis: Cq) for a multiplex qPCR assay of feline parvovirus (FPV), feline bocavirus 1 (FBoV-1), feline kobuvirus (FeKoV) and feline astrovirus (FeAstV). Each virus plasmid is from 10^8^ to 10^2^ copies/μL. **(B)** Standard curve formulae in the multiplex qPCR for FPV, FBoV-1, FeKoV, and FeAstV.

The results showed that multiplex qPCR could efficiently detect all target genes of the four viruses with high correlation values. All standard curves showed good correlation coefficients and amplification effects, with the correlation coefficients of FBoV-1 (R^2^ = 0.9985; Eff% = 99), FeAstV (R^2^ = 0.9967; Eff% = 96), FeKoV (R^2^ = 0.9976; Eff% = 98), and FPV (R^2^ = 0.9969; Eff% = 87), as shown in Figure 2.

### Specificity of the multiplex qPCR

**Figure 3:**
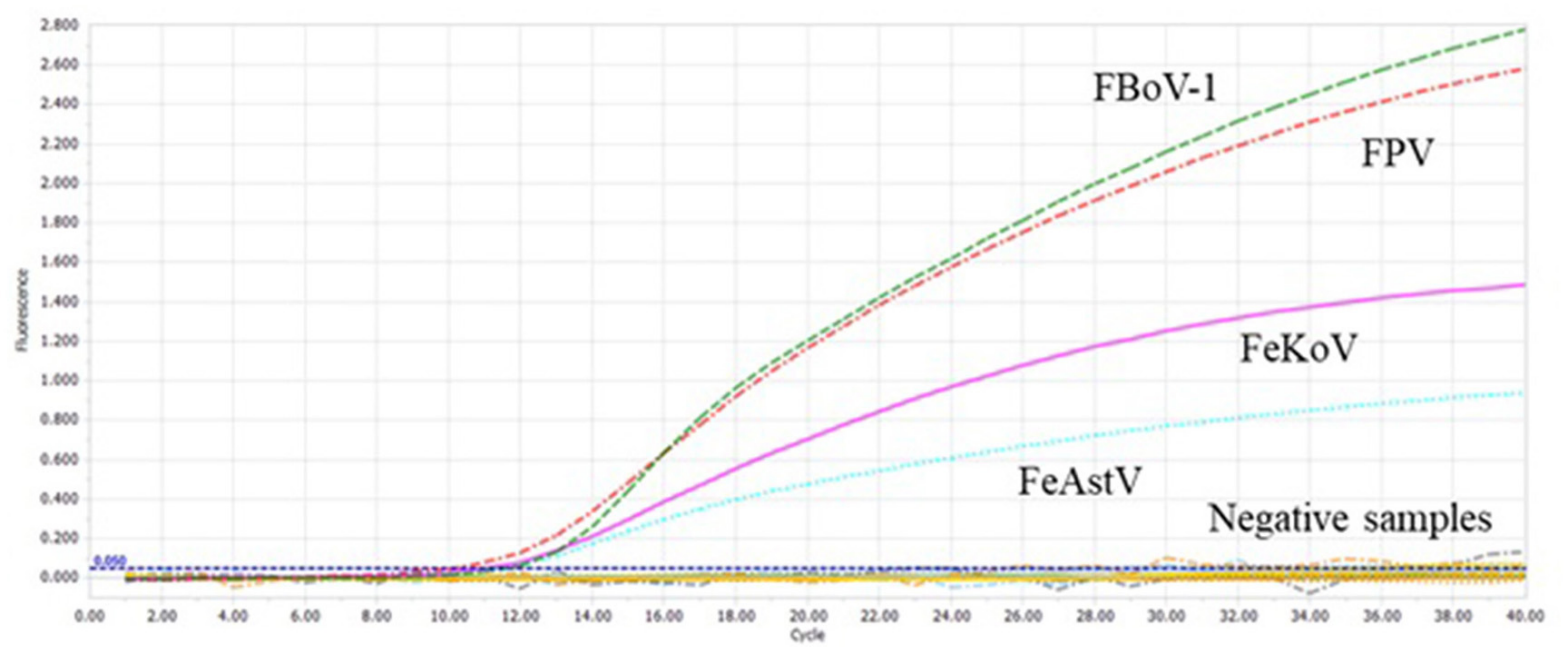
The specificity of the multiplex qPCR. Feline bocavirus 1 (FBoV-1), feline parvovirus (FPV), feline kobuvirus (FeKoV), and feline astrovirus (FeAstV) showed specific amplification curves in the multiplex qPCR assay. Other samples include feline bocavirus 2 (FBoV-2), FBoV-3, feline coronavirus (FCoV), feline chaphamaparvovirus (FeChPV), and nuclease-free water.

FPV, FBoV-1, FBoV-2, FBoV-3, FeAstV, FeKoV, FCoV, FeChPV, and nuclease-free water were amplified to detect the specificity of the multiplex qPCR technology developed in this research. ddH_2_O was used as the negative control, and only the detection result of the target virus was positive (Figure 3), indicating that this method was highly specific.

### Repeatability of the multiplex qPCR

To verify the repeatability of this experimental method, three different researchers conducted detection experiments at different times, and all data were collected for analysis. Both intra- and inter-assay analyses showed that the multiplex qPCR was highly repeatable (Table 3).

**Table 3:** The repeatability of the multiplex qPCR.

	**Intra-assay**				**Inter-assay**			
**Templates (copies/**μ**L)**	**FPV Cq values**			**CV%**	**FPV Cq values**			**CV%**
10^8^	10.12	10.52	10.27	1.96	10.33	12.08	11.36	7.80
10^7^	14.16	14.08	15	3.54	14.57	15.11	14.93	1.86
10^6^	16.64	17.21	17.59	2.79	17.15	18.76	17.8	4.53
10^5^	20.69	20.47	21.03	1.36	20.73	21.96	20.47	3.78
10^4^	24.81	24.25	25.11	1.77	24.76	25.44	24.83	1.50
10^3^	28.01	28.55	27.96	1.16	28.17	29.68	28.71	2.65
10^2^	32.49	31.32	31.50	1.98	31.76	32.77	31.54	2.05
**Templates (copies/**μ**L)**	**FBoV-1 Cq value**			**CV%**	**FBoV-1 Cq value**			**CV%**
10^8^	11.59	11.82	11.76	1.02	11.72	11.65	11.35	1.71
10^7^	15.3	15.39	15.75	1.54	15.60	14.86	14.77	3.02
10^6^	18.04	18.74	18.44	1.91	18.60	18.12	17.84	2.12
10^5^	21.37	21.84	22.02	1.54	21.74	21.35	20.68	2.53
10^4^	25	24.76	25.34	1.16	25.03	25.56	24.49	2.14
10^3^	28	28.44	28.69	1.23	28.38	27.99	28.04	0.75
10^2^	32.02	32.53	32.51	0.89	32.35	31.68	32.04	1.05
**Templates (copies/**μ**L)**	**FeKoV Cq value**			**CV%**	**FeKoVCq value**			**CV%**
10^8^	10.99	11.26	11.24	1.35	11.16	12.25	11.97	4.78
10^7^	14.98	14.85	15.12	0.90	14.98	15.33	14.66	2.24
10^6^	17.76	18.07	17.98	0.89	17.94	18.95	17.84	3.37
10^5^	21	21.12	21.43	1.05	21.18	21.98	21.05	2.35
10^4^	25.11	24.88	25.03	0.47	25.01	25.48	24.17	2.67
10^3^	28.35	28.24	28.86	1.16	28.48	28.99	28.15	1.48
10^2^	31.03	31.46	32.11	1.72	31.53	32.46	32.51	1.71
**Templates (copies/**μ**L)**	**FeAstV Cq value**			**CV%**	**FeAstV Cq value**			**CV%**
10^8^	11.4	11.32	11.55	1.02	11.42	10.83	11.65	3.75
10^7^	15.33	14.88	14.96	1.59	15.06	13.97	15.24	4.65
10^6^	18.34	18.21	18.84	1.80	18.46	17.8	17.84	2.06
10^5^	21.61	22.01	21.79	0.92	21.80	21.23	21.05	1.84
10^4^	25.52	25.55	25.31	0.51	25.46	25.17	24.36	2.28
10^3^	27.88	28.71	28.36	1.47	28.32	29.54	28.11	2.70
10^2^	32.53	32.4	31.99	0.87	32.31	33.75	31.84	3.05

### Sensitivity of the multiplex qPCR

To determine the sensitivity of this method, four viral standard plasmids from 10^8^ to 10^1^ copies/μL were used for detection under optimized reaction conditions. The results showed that the sample detection rate was low when the plasmid was 10^1^ copies/μL; the detailed data are shown in Table 4. It was unstable and did not meet the requirements of high repeatability and stability. For plasmids of 100 copies/μL, each virus was detected in every assay. Therefore, we believe that the minimum detection limit of this assay is 100 copies/μL.

**Table 4:** Sensitivity of the multiplex qPCR assay.

**Templates**	**Copies/**	**Total**	**Positive**	**Rate**	**95% confidence**
	**μL**		**detection**	**(%)**	**region**
FPV	100	45	45	100	√
	10	45	37	82.2	×
FBoV-1	100	45	45	100	√
	10	45	34	75.6	×
FeKoV	100	45	45	100	√
	10	45	26	57.8	×
FeAstV	100	45	45	100	√
	10	45	11	24.4	×

### Agreement of the multiplex qPCR with sequencing

The method developed in this study was used to detect fecal samples from 135 cats with diarrhea symptoms, and the positive samples were identified via sequencing by the General Biology Company (Anhui, China).

### Infection rate of the clinical samples

**Figure 4:**
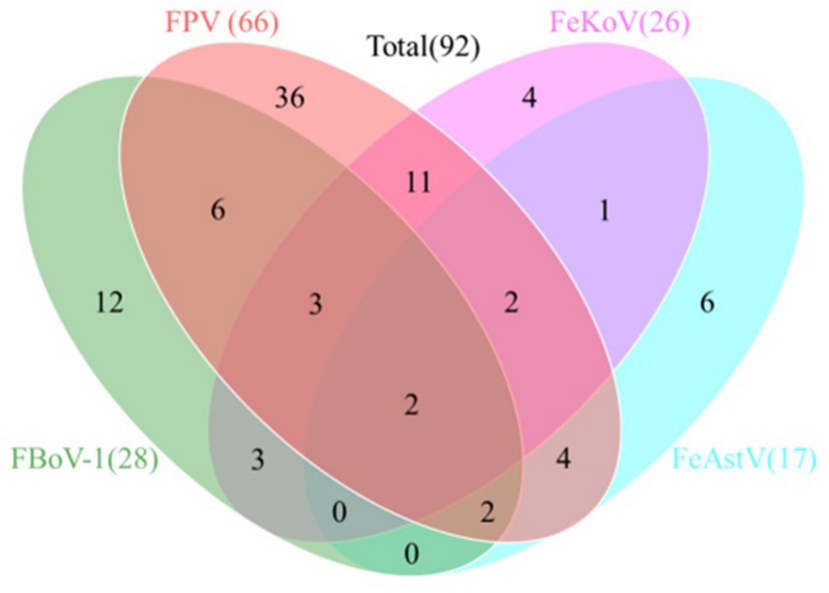
Co-infection analysis of detected feline viruses. Total 135 clinical feline fecal samples collected from 2018 and 2021 were analyzed by the multiplex real-time qPCR assay. A total of 92 samples were detected as positive. The Venn diagram shows the number of samples infected by either single virus or multiple viruses.

Table 5 shows that the total detection rate of the four viruses in all samples was 68.15% (92/135), and the FPV positive rate was the highest with 48.89% (66/135), while the positive rates of FBoV-1, FeAstV, and FeKoV were 20.74% (28/135), 12.59% (17/135), and 19.26% (26/135), respectively. The sum rate of co-infection among the viruses was 25.19% (34/135). Two samples tested positive for all four viruses, with an infection rate of 1.48% (2/135), as shown in Figure 4.

**Table 5:** Infection rates from the clinical feline stool samples.

**Virus**	**FPV**	**FBoV-1**	**FeKoV**	**FeAstV**	**Positive virus**
Total infection rate	(66/135) 48.89%	(28/135) 20.74%	(26/135) 19.26%	(17/135) 12.59%	(92/135) 68.15%
Co-infection rate	(30/135) 22.22%	(16/135) 11.85%	(22/135) 16.30%	(11/135) 8.15%	(34/135) 25.19%
Separate infection rate	(36/135) 26.67%	(12/135) 8.89%	(4/135) 2.96%	(6/135) 4.44%	(58/135) 42.96%
Double infection rate	(21/135) 15.56%	(9/135) 6.67%	(15/135) 11.11%	(5/135) 3.70%	(25/135) 18.52%
Triple infection rate	(7/135) 5.19%	(5/135) 3.70%	(5/135) 3.70%	(4/135) 2.96%	(7/135) 5.19%
Quadruple infection rate	(2/135) 1.48%	(2/135) 1.48%	(2/135) 1.48%	(2/135) 1.48%	(2/135) 1.48%

## Discussion

Feline viral diarrhea poses a serious threat to the health of domestic cats. Before 2018, FBoV-1, FeAstV, and FeKoV had not been reported in mainland China. FPV and FCoV are important factors for the clinical diagnosis of viral diarrhea in domestic cats (25, 26). As novel feline diarrheal viruses are gradually being reported and spreading in mainland China, the current clinical detection method for viral diarrhea in cats needs to be updated (3, 7, 15). Of the four viruses selected for this study, FPV is one of the most widespread and harmful, usually infecting young and unvaccinated cats. However, a small number FPV infections in vaccinated cats still result in immune failure (27). The pathology and viral proclivity of FBoV-1 infection have been described in cases related to outbreaks of hemorrhagic enteritis in domestic cats (4). Currently, FBoV-1 is more widespread in mainland China than FBoV-2 and FBoV-3. The results of comparison shows that ORF2 protein of FeAstV is more homologous to the human AstV rather than that of other mammals. It suggests the possibility of relatively recent hybridization (28). A FeAstV-like astrovirus was found in a symptomatic child, suggesting the possibility of direct cross-species infection (13). FeKoV is reported to be positively correlated with feline diarrhea in several countries (7, 19, 20). As these viruses have a high probability of mixed infection, high pathogenicity, and similar clinical symptoms, it is important to monitor these viruses in clinical diagnosis. Molecular diagnostic tools are urgently needed for the efficient and accurate diagnosis of these viruses. In recent years, qPCR detection methods and traditional PCR detection methods for FPV (22), FBoV-1 (23), and FeAstV (24) have been developed, but no RT-qPCR is available for detection of FeKoV. There is currently no method to detect all four viruses in a single tube; therefore, the detection methods for these related viruses urgently need to be updated.

qPCR based on TaqMan probes is a rapid, highly specific, sensitive, and reproducible tool for virus identification and detection (29). In this study, we established a multiplex qPCR method based on TaqMan probes for the simultaneous detection and identification of FPV, FBoV-1, FeAstV, and FeKoV in cats with diarrhea. Multiplex qPCR has the advantages of high throughput, sensitivity, and detection efficiency. However, in multiplex qPCR detection, multiple oligonucleotides are present in the reaction system at the same time, increasing the possibility of non-specific amplification. Therefore, primer and probe designs are more rigorous than those of single qPCR (30).

In this study, the minimum detection limit of single qPCR for each virus was 10 templates. However, the detection limit of each target gene in the multiplex qPCR was ~100 copies. We speculate that the detection sensitivity of multi-detection is reduced because of competition among primers, probes, templates, and reagents of different viruses compared to single detection. The correlation coefficients of all the reactions were higher than 0.995, indicating an extremely high correlation. The results of the inter- and intra-assay analyses showed that the experiment had high repeatability. Because of the high sensitivity of the probe method, our technique increases the detection limit of each virus from 10^4^ to 10^2^ compared with the mPCR constructed by Zhang et al. (2) and it also allows the detection of FeKoV.

Our clinical detection method showed that viral infections were prevalent in cats with diarrhea (68.15%, 92/135), and that the infection rate of FPV was high (48.89%, 66/135). Viral co-infections were common in diarrheal cats (25.19%, 34/135), and FPV was present in 30 out of the 34 co-infection positive samples. Two samples were positive for FPV, FBoV-1, FeAstV, and FeKoV co-infections (1.48%, 2/135). The results showed that FPV remains the dominant virus strain in diarrheal cats in East and South China, and that co-infections of FBoV-1 (20.74%, 28/135), FeAstV (12.59%, 17/135), and FeKoV (19.26%, 26/135) were common. Our findings suggest that other diarrhea-associated viruses cannot be ruled out besides FPV in the cats with diarrhea. This study provides a powerful tool for the diagnosis of related viruses and improves the efficiency of the surveillance of these novel feline viruses.

Since clinical samples were collected from diarrheal cats in animal hospitals and animal rescue stations from July 2018 to September 2021, the specific age and breed data of some disease-affected samples were vague, and thus, the statistical analysis was complicated and difficult. Hence, we decided not to perform statistical analysis. Instead, we collected data on the infection status of the four viruses in the feces of cats with diarrhea and performed simple statistics and descriptions of the infection rates of the four viruses in cats with diarrhea. More detailed statistics and descriptions are warranted in future in-depth research. Moreover, detailed studies on parasites and bacteria associated with cats will further enhance the value of this research.

In summary, we established an efficient and accurate detection technology that can effectively detect and prevent diseases. The advantages of multiplex qPCR including shorter time, cost efficiency, high throughput, and quantitative analysis, greatly increase the upper limit of detection methods. However, it is still more difficult and challenging than the development of a single qPCR assay. To our knowledge, this is the first multiplex qPCR assay to simultaneously detect and identify FPV, FBoV-1, FeAstV, and FeKoV. We first established a detection method for FeKoV and added it to a common assay for other feline diarrhea-associated viruses. This method has high sensitivity, specificity, and repeatability, which can improve the detection of clinical co-infection, be more convenient, save materials, and reduce workload. It represents a good tool for the clinical diagnosis and epidemiological analysis of FPV, FBoV-1, FeAstV, and FeKoV.

## Data availability statement

The datasets presented in this study can be found in online repositories. The names of the repository/repositories and accession number(s) can be found in the article/supplementary material.

## Author contributions

JZ and YW conceived the study. JZ performed experiments, wrote the manuscripts, and revised it. JY record the experimental data. YM, XX, RW, HW, XL, FX, and JW collected clinical samples. All authors have read and approved the final version of the manuscript.

## Conflict of interest

The authors declare that the research was conducted in the absence of any commercial or financial relationships that could be construed as a potential conflict of interest.

## Publisher's note

All claims expressed in this article are solely those of the authors and do not necessarily represent those of their affiliated organizations, or those of the publisher, the editors and the reviewers. Any product that may be evaluated in this article, or claim that may be made by its manufacturer, is not guaranteed or endorsed by the publisher.
